# The Empusa code generator and its application to GBOL, an extendable ontology for genome annotation

**DOI:** 10.1038/s41597-019-0263-7

**Published:** 2019-11-04

**Authors:** Jesse C. J. van Dam, Jasper J. Koehorst, Jon Olav Vik, Vitor A. P. Martins dos Santos, Peter J. Schaap, Maria Suarez-Diez

**Affiliations:** 1 Laboratory of Systems and Synthetic Biology, Wageningen University & Research, Wageningen, 6708 WE The Netherlands; 2 Centre for Integrative Genetics (CIGENE), Department of Animal and Aquacultural Sciences (IHA), Faculty of Life Sciences (BIOVIT), Norwegian University of Life Sciences (NMBU), PO Box 5003, Ås, Norway

**Keywords:** Genome, Software

## Abstract

The RDF data model facilitates integration of diverse data available in structured and semi-structured formats. To obtain a coherent RDF graph the chosen ontology must be consistently applied. However, addition of new diverse data causes the ontology to evolve, which could lead to accumulation of unintended erroneous composites. Thus, there is a need for a gate keeping system that compares the intended content described in the ontology with the actual content of the resource. The Empusa code generator facilitates creation of composite RDF resources from disparate sources. Empusa can convert a schema into an associated application programming interface (API), that can be used to perform data consistency checks and generates Markdown documentation to make persistent URLs resolvable. Using Empusa consistency is ensured within and between the ontology and the content of the resource. As an illustration of the potential of Empusa, we present the Genome Biology Ontology Language (GBOL). GBOL uses and extends current ontologies to provide a formal representation of genomic entities, along with their properties, relations and provenance.

## Introduction

Semantic Web technologies provide information retrieval and management systems to integrate heterogeneous data from disparate sources^1^. The RDF data model is a W3C standard for storage of information in the form of self-descriptive Subject, Predicate and Object triples that can be linked in an RDF-graph^2,3^. The use of retrievable controlled vocabularies enables integration of heterogeneous diverse data from different sources in a single repository and SPARQL can be used to query the so generated resources^4,5^.

By themselves, RDF graphs have no predefined structure nor a schema, and the structure of an RDF resource can vary as new triples are added. Therefore, a formal definition of the relations among the terms, called an ontology, is required to efficiently retrieve linked information from these resources. Structural information can be encoded using Web Ontology Language (OWL) files^6^. RDFS is another, related, standard to define the structure of an RDF resource^7^. In this standard, each object can be defined as an instance of a class and each link as the realisation of a property. Shape Expressions (ShEx) is a standard to describe, validate and transform RDF data. One of the goals of this standard is to create an easy to read language for the validation of instance data^8–10^.

In the development of RDF resources, transformation of existing data into the RDF data model is often a source of errors such as typing errors in the predicates, instances with missing attributes, non-unique Internationalized Resource Identifiers (IRIs), or no type defined, among others. In previous work, we developed RDF2Graph, a tool to automatically recover the structure of an RDF resource and to generate a visualisation, ShEx file and/or an OWL ontology thereof^11^. Application of RDF2Graph to resources providing data in the RDF data model in the life sciences domain such as Reactome, ChEBI, UniProt, or those transformed by the Bio2RDF project^12–16^ showed mismatches between the retrieved data structure and the one described in the OWL definition of the particular resource. The main reason for this lack of consistency is the flexibility provided by RDF: the data graph is a free format, the ontology defines the structure but does not enforce it. Tools that use the RDF data model as means to store their output may therefore be essential to unlock the potential of these technologies in the life sciences. Their development would be greatly facilitated by supporting tools able to read an ontology definition and generate code that can be used for data generation, export and validation.

**Fig. 1:**
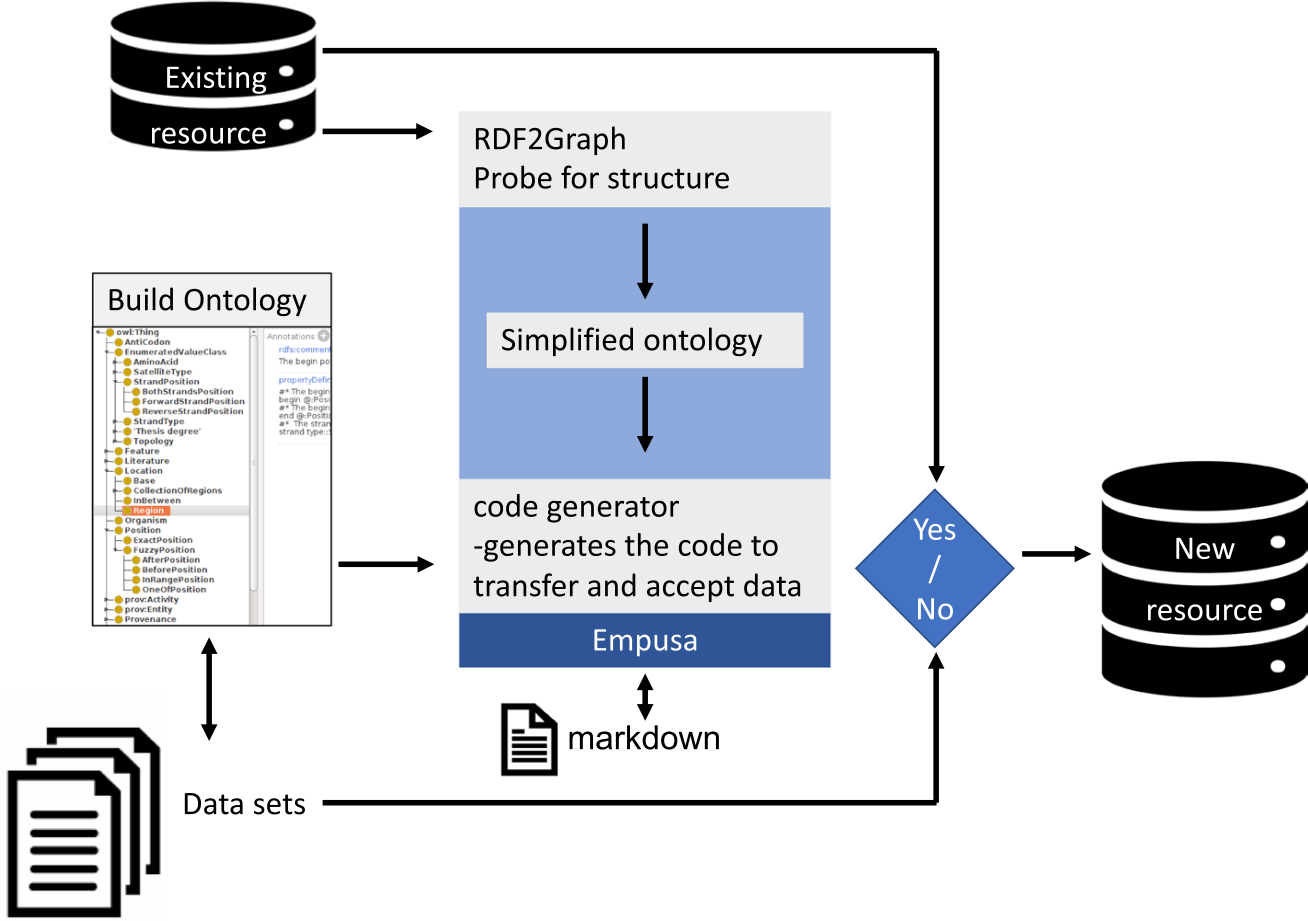
Simplified overview of the workflow to manage consistent integration of new diverse data with existing resources. Empusa enables error control as it compares the intended content, described in the ontology, with the actual content of the resource. For this, Empusa checks whether or not *Subjects* and *Objects* have the properties that the ontology demands. Empusa builds upon RDF2Graph^11^, a tool to automatically recover the structure of an RDF resource, to generate a visualisation, ShEx file, and/or an OWL ontology thereof.

Here we present *Empusa*. Empusa has been developed to facilitate the creation of RDF resources, which are validated upon creation (Fig. 1). Empusa can convert a schema into an associated application programming interface (API), that can be used in Java and R to perform data consistency checks and generates Markdown documentation to make persistent URLs resolvable^17^.

## Results

The Empusa code generator has been applied to the Genome Biology Ontology Language (GBOL), that uses and extends current ontologies to provide a formal representation of genomic entities. Advances in sequencing technologies have turned genomics into a data-rich scientific discipline that relies on automated annotation algorithms to supplement to manual annotation^18,19^. The GenBank format is currently used for sharing genome annotation. However, tradeoffs between simplicity, human readability and representational power, left little support for interoperability, i.e. the ability of computer systems to directly make use of information.

Large scale comparative analysis of genome data requires a framework such as SAPP^20^ able to accommodate the various types of annotations (e.g. gene and protein domain predictions) consistently interlinked with the supporting statistical evidence so that data becomes FAIR^21^. Using standard tools, SAPP automatically annotates genome sequences. In SAPP, GBOL and the GBOL stack of enforcing tools are used to describe and link genome annotations with provenance.

Empusa was developed primarily to help develop ontologies focusing on their function as a database schema for RDF resources. The design principles *modularity*, *human readability*, and *annotation* are followed. These principles are reflected in GBOL as described below.

### Modularity

The number of classes in the main class tree is kept as small as possible and elements within the data are described with attributes when possible. Furthermore, classes are included in the main class tree only when there are unique properties in a class or in one of the sibling classes. This approach ensures that sub-ontologies can be managed as separate entities within the main ontology and that we can use existing ontologies. As an example the class *RegulationSite* has an attribute *regulatoryClass*, which denotes the type of regulation with a separate set of classes of which all are instances of the *regulatoryClass*.

To further simplify the ontology, every attribute is defined as a direct property within the class that links to either a string, an integer, another object or a class in an enumeration set. For each class in which the attribute is used, an ‘all values from’ axiom is used, with an optional minimal and/or maximal cardinality constraint. The ‘all values from’ axiom enforces all referenced objects to be of the expected type, which is not the case with the ‘some values of’ axiom and therefore we excluded the use of the ‘some values of’ axiom. This approach is fundamentally different from the principle used in the Sequence ontology^22^, in which attributes are defined using the ‘has quality’ property in combination with the ‘some values of’ axiom that references to a class.

### Human readability

All names within the ontology adhere to a set of basic principles to increase (human) readability of the ontology. All class names represent the underlying biological concept as closely as possible avoiding the use of unreadable numbers. All classes start with uppercase whereas properties start with lowercase. All words are spelled out, and white spaces are left out of the names, instead the next word starts with uppercase. In this way, the class ‘exact position’ becomes ‘ExactPosition’ and the property ‘regulatory class’ becomes ‘regulatoryClass’. Furthermore, where possible, the names are shortened with abbreviations, as long as they remain understandable for a human reader (e.g. XRef instead of CrossReference).

### Annotation

All classes and terms within the ontology are annotated with a short definition; an optional comment with additional usage information; an optional editorial comment relating to the development of the ontology itself; an optional *ddbj* label indicating the presence in the GenBank standard; and an optional SKOS^23^ exact match to relate classes to terms in existing ontologies.

#### GBOL structure

GBOL provides the means to consistently describe computationally inferred genome annotations of biological objects typically found in a genome sequence annotation data file in public repositories. Additionally, GBOL can describe the data provenance of extracted genetic information.

**Fig. 2:**
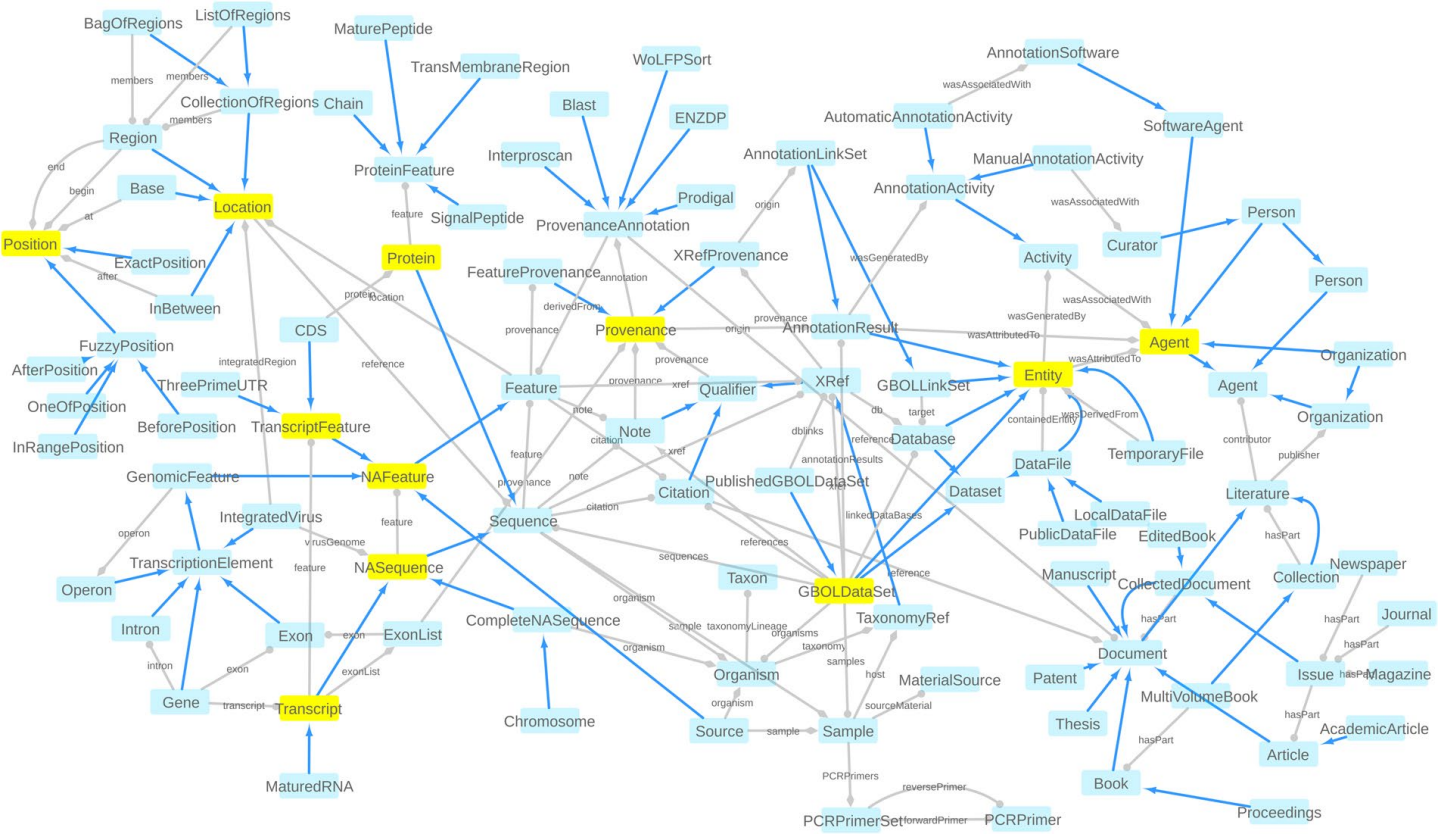
The GBOL ontology structure. Nodes represent types. Blue edges represent *subClassOf* relationships whereas grey edges represent unique type links. A unique type link is defined as a unique tuple: type of subject, predicate, (data)type of object. Arrow heads indicate the forward multiplicity of the unique type links: 0..1 and 1..1 multiplicities are indicated by diamonds; 0..N and 1..N multiplicities are indicated by circles. Neighbourhood of nodes marked in yellow is further expanded in Fig. 3.

An overview of the structure of GBOL is shown in Fig. 2. The ontology contains 251 classes that can be categorized into 6 broad domains (Table 1). In GBOL, sequences have features, which in turn have genomic locations on the sequence. The authority of this relationship is derived from the data provenance that captures both the statistical basis of each individual annotation (element-wise provenance) as well as the programs and parameters used for the complete set of sequences under study (dataset-wise provenance). All annotations for a given sequence can be packed into a single entity called a document.

**Table 1:** Overview of domains, classes and properties described by the the GBOL ontology.

Sub domain	Classes	Properties	Value sets
Genomic locations	16	17	1
**Genes**			
transcripts and features	114	133	17
Document structure	27	107	7
Dataset-wise provenance	22	54	0
Element-wise provenance	5	9	0
BIBO	59	90	2

Note that some properties might be in multiple sub domains.

### Key GBOL classes

Common elements in genome annotation include different classes of DNA molecules, transcripts, proteins, exons, protein domains and other functional annotations. In the following we summarise two key classes of the ontology: Genomic locations and Provenance (see Table 1). Extensive descriptions for each class and element in GBOL can be found in the Empusa generated documentation available at http://gbol.life.

#### Genomic locations

**Fig. 3:**
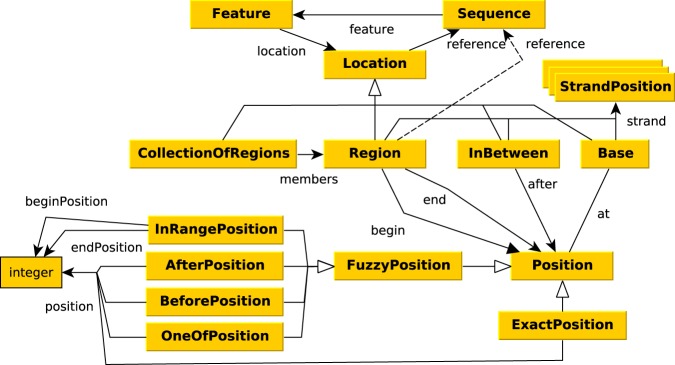
Graphical view of the GBOL ontology for genomic locations. An explanation of the classes is provided in the main text.

Annotation of genomic location is inspired by FALDO ontology^24^. Genomic locations of all features in GBOL is captured with the *Location*, *Position* and *StrandPosition* classes, which are represented in Fig. 3. The *Location* and its subclasses together with the *StrandPosition* define an interval on the Sequence, whereas *Position* defines a single position in a sequence. A location can be either: (*i*) A region which has begin and end positions; (*ii*) A collection of regions (ordered or unordered); (*iii*) A single base at a given position; or (*iv*) an *InBetween* location denoting a location between two bases after the base of which the position is given. Each region, base and in-between location can be defined to be located on the forward, reverse or both strands, although no strand should be specified if the sequence is a single stranded DNA sequence or a protein sequence. It should be noted that elements of a collection of regions can be located on different sequences. This can be used to encode cases in which an otherwise indistinguishable genetic element is located on multiple chromosomes.

Exactly known positions can be indicated using the *ExactPosition* class containing the *position* property. Otherwise a not exactly known position, also called fuzzy position, can be indicated using either the *BeforePosition* class containing the *position* property, the *AfterPosition* class containing the *position* property, the *InRangePosition* class containing the *beginPosition* and *endPosition* properties or the *OneOfPosition* class containing multiple *position* properties.

#### Provenance

Three types of provenance can be distinguished. Metadata refers to the ownership of the samples, the biological origin, culture conditions etc. All data within a single data collection stored in GBOL is based on the *GBOLDataSet*, which holds the metadata, composed among other of references to all included samples, sequences, organisms, annotation results and linked databases. Dataset- and element- wise provenance pertain to the annotation process.

#### Dataset-wise provenance

Storage of the dataset-wise provenance is based on the PROV-O ontology^25^ in which the *Activity* class is central. Within GBOL, each activity is an annotation activity. An automatic annotation must be associated with a software agent, a set of parameters and the corresponding input and/or output files. Manual curation must be associated with a curator.

#### Element-wise provenance

Each annotation tool generates its own evidence statements, often embedded in a statistical framework, characteristic of the algorithmic approach taken, such as p-values, bit scores, matching regions or any other scoring system. This element-wise provenance of all the annotation in GBOL is captured per property per feature with the *FeatureProvenance*. To store tool specific confidence scores, subclasses of the *ProvenanceAnnotation* class can be created. Some example classes include *Blast*, *HMM* and *SignalP* associated with the output of corresponding tools^26–28^. However, these classes are not part of the GBOL ontology itself.

### Extensibility and link to existing ontologies

A challenge in ontology development is consistent incorporation of existing ontologies. Empusa ensures correct usage of existing ontologies. Empusa leads to the development of an API that can be used to perform data consistency checks between the existing ontologies and the one under development.

In the development of GBOL we made extensive use of this gate keeping function, as ontologies already exist for various aspects of biology^29^. In this manner we were able to embed GBOL in the set of existing ontologies such as FALDO^24^, PROV-O^25^, SO^22^, SBOL^30^, BIBO^31^, WikiData^32^, FOAF^33^, Gene ontology (GO)^34^ and the Evidence ontology^35^. For instance, whenever applicable, we added a cross-link to exact matching terms within the FALDO, SO and SBOL ontologies^22^, and identification of persons and institutions is done through the FOAF ontology, and BIBO is used to identify publications.

Moreover, the use of Empusa turns GBOL into and extendable ontology: although GBOL has been primarily designed to handle genomic annotation, in the future it can be extended to host other ~ omics data types as proteomics and transcriptomics. The modular design ensures that other ontologies can be incorporated and managed as separate entities. For instance, the majority of the feature and sequence classes within GBOL can be connected with those from the SO. The major difference between GBOL and SO is that SO has been defined as vocabulary of terms related to genetic elements, whereas the GBOL classes have been designed to describe genetic annotation and elements located on a sequence and is inspired on the principles of the GenBank format. However, still a number of features in the SO are not currently available in GBOL and future work should focus on including them. Another possible extension would be to link to Minimum Information Standards like MIGS and extensions thereof (MIMARKS, MIxS)^36,37^ and cross domain experiment reporting standards like ISA-tab^38^. Other possible extensions relate to the development of the sub-ontologies GBOL links to. For instance, BIBO is used to store information on literature references, however the OWL ontology file of BIBO has to be further improved, as it does not specify to which classes all of the properties should belong.

## Discussion

Empusa was developed primarily for ontologies focusing on their function as a schema for RDF resources. This is achieved through the design principles modularity, human readability, and annotation. Data sharing in the life sciences requires the use of concepts and terms that can be matched across resources. This matching requires ontologies, so that a concept is defined within the context of other concepts. Another requirement is that data are stored in a universal representation that can be readily interlinked with other resources and data sets. Using RDF, this is achieved by representing the data as a graph in which the nodes represent instances of concepts and the links represent properties that describe the instance from which they originate. The OWL standard can be used to create ontologies to define concepts and to link these to the associated terms while ShEx (and SHACL) standards can be used to validate the instance data, which ensures that the structure of the data follows the rules defined within the ontology. An example of instance data validation would be to verify that a protein has one and only one amino acid sequence associated to it.

The growth of semantic web technologies has led to using the technologies developed for concept matching to link data. This might lead to mismatches between design principles and effective usage. For instance, the GO and SO ontologies. were developed to characterize gene function and nucleotide sequences respectively. Currently they are used in resources such as UniProt^16^ to unambiguously refer to biological concepts. However, these ontologies are not suited to store all the information of the objects themselves within an inter-linkable and reusable semantic data graph. For example, SO can be used to indicate that a part (indicated with FALDO) of a nucleotide sequence corresponds to a silencing RNA. However SO cannot be used to describe all the properties of the silencing RNA as it cannot describe what are the targets of the silencing RNA. Likewise, the GO ontology can be used to annotate genes with a given biological or molecular function, however it cannot be used to describe a biochemical reaction not included in the GO ontology.

GBOL was developed to consistently capture annotation data generated by SAPP^20^. Initial versions of the GBOL ontology have been used for comparative genomics analysis and to show that protein domain architectures are well suited for comparative functional genomics^39^. GBOL was used to hold the data underlying the large scale comparison of *Pseudomonas* genomes (432 species)^40^ to identify key characteristics of this genus such as the sizes of the core and pan genomes and to clarify the link between gene essentiality and persistence. The comparison 80 mycoplasma genomes was also enabled by GBOL^41^ and shed light on host specificity. GBOL can also hold eukaryotic genome data as illustrated in^42^. Currently the GBOL stack is being used in various collaborative projects to handle genomic data of organisms across all domains of life (DigiSal, INFECT, MycoSynVac, EmPowerPutida).

GBOL enables interoperable genome annotation, as it deploys and extends existing ontologies to represent genomic entities, their properties and relations and associated provenance. The GBOL Stack, generated using the Empusa code generator, provides a framework to enforce consistent and correct usage of GBOL. The semantic basis and the integration of provenance enables FAIR genome annotations, thereby enabling large scale analysis of heterogeneous biological data and unlocking the potential of functional genome annotation.

Empusa was developed primarily to help develop ontologies focusing on their function as a database schema for RDF resources. The design principles *modularity*, *human readability*, and *annotation* ensure that the so generated ontology can be easily extended^43^. This allows users to browse the complete ontology intuitively. The Java and R APIs can be used to verify the consistency of the resource using ShEx (a use case is presented in the Empusa documentation).

Modularity and readability also ease the expansion of GBOL. We separated the sub-ontologies (value set) definition from the definition of the classes that have properties associated to them. In this way, value sets are defined under the *EnumeratedValue* class and can be seen as sub-ontologies. This ensures that a value set can evolve into a full ontology, and a class/sub-class structure can be defined for these elements. For instance, in GBOL, a value set is be defined for nucleic acids that contains *adenine*, *cytosine*, *guanine*, *thiamine*, *uracil* and *inosine*. In the future this could be extended with alternative forms. However, inclusion of all alternative forms and modifications would cause the complexity of the ontology to explode. Thus, instead of adding values to the value set, a class with properties describing the chemical representation could be added.

The GBOL stack contains over 80.000 lines of R and Java code, OWL and ShEx definition files, and documentation files (mkdocs format). Generating such a large amount of code would entail 1 year of manual work (considering an efficiency of 50 lines per hour)^44^. Moreover, during the development of the GBOL ontology countless updates were made to correctly encapsulate all the data and associated provenance. Most of these updates were based on insights gained through the data encoding process. Manually updating the code, without using the supporting Empusa tool, would have entailed so much work that it would still be an on-going process. Thus, the Empusa code generator can serve to reduce the time (and costs) associated to development of ontologies and tools.

In conclusion, the Empusa code generator can be used to develop new ontologies combined with automatic generation of API and documentation. This reduces the complexity and time to extend and develop ontologies and tools able to exploit the full potential of Semantic Web technologies for heterogeneous data integration. Moreover, Empusa enables the validation of the generated resources and the verification of the consistency of the exported data thereby bridging the gap between the intended and the actual content of RDF resources.

## Methods

The input definition of Empusa is a combination between OWL and a simplified version of ShEx, which can be edited within Protégé^45^. OWL makes the open world assumption and it is not suitable for closed world structure checking. In case of a class that states that an associated property is obligatory, an instance not having the corresponding link would be wrong, however an OWL processor would not report this mismatch as the corresponding link would be assumed to exist in an open world. ShEx was designed for data conformance tests under the closed world interpretation, so that the absence of the obligatory link will be reported^46^. ShEx provides complementary benefits and we combine OWL and ShEx to enable standardized restrictions implemented through ShEx. At the end of the process, Empusa outputs OWL and ShEx files conforming to their respective standards. The classes are defined in OWL, whereas the properties are defined in each class under the annotation property *propertyDefinitions* encoded within a simplified format of the ShEx standard. Additionally predefined value sets can be defined by adding a subclass to the *EnumeratedValueClass*. For instance a *FileType* can only be one element of a predefined list (e.g. CSV,TXT,TSV).

**Fig. 4:**
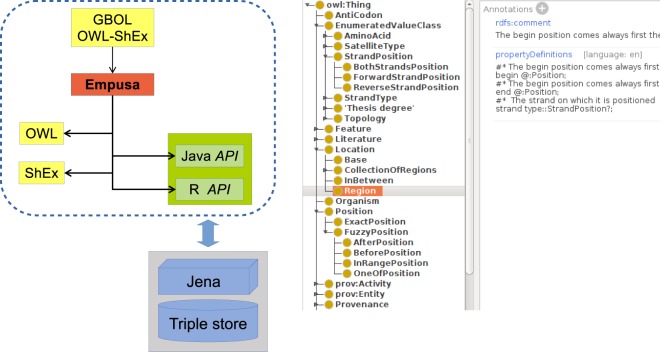
Empusa file definition. *Left*: The input definition file (combining OWL and SHeX) is used to provide an ontology (here the GBOL ontology is used as example). Empusa generates as output: an OWL file definition, a ShEx file that can be used for instance validation, the corresponding documentation in Markdown format, and R and Java APIs. *Right* Example input file. Properties within a class can be defined with the *propertyDefinitons* annotation property. As an example, the *Region* class has been highlighted. Value sets (sub-ontologies) can be defined under the *EnumeratedValueClass* class, for example the *StrandPosition* value set.

The RDFS standard is used to define the *subClassOf* relationships between the classes, whereas the ShEx standard is used to define the properties of each class. Properties of the class are defined through the annotation property *propertyDefinitions* as shown in Fig. 4. For each property the multiplicity and the expected type of the target value can be defined. The multiplicity can either be: *0..1* indicating that the property is optional and at most one reference is allowed; *1..1* indicating that one and only reference is allowed; *0..N* for optional properties with multiple allowed references; and *1..N* for properties that must have at least one reference. The ‘=’ and ‘~’ sign can be used to define the references to be stored as an ordered or numbered list to ensure that the elements are numbered. Target value types can also be defined. The type of the target value can be either: A simple value (String, Integer or Double, among others); Another class (for example a Protein); Or an IRI, referencing an external resource or ontology or to a sub-ontology (value set). Within the ontology, value sets are be defined as subclasses under the *EnumeratedValue* class. In this way every sub-class of *EnumeratedValue* can be expanded as a sub-ontology. All subsequent sub-classes are elements of the sub-ontology of which it is sub-classed from.

The Empusa code generator uses this definition to generate: (*i*) An OWL file definition. It should be noted that the OWL file definition is generated as it remains general consensus within the field of semantics that these files are created for each ontology. (*ii*) A full ShEx file that can be used to validate a data set containing information that is encoded with the ontology. (*iii*) An R and Java API, which one can use to generate the data with the encoding of the defined ontology. This API ensures that the multiplicities and referenced types are correct and prevents many errors in the data export. (*iv*) A full documentation of the ontology based on *mkdocs*. The *rdfs:label* and *skos:description* properties can be used within the ontology to add a description about the classes and a comment line above each property definition in the simplified ShEx definition and can be used to add a description to each property.

Regarding GBOL, storage of the genomic location is inspired by FALDO, although several elements had to be modified e.g. to account for features that start and end on different sequences. Differences include: (*i*) *StrandPosition* is not subclassed from *Position*. Instead, an additional property is added to the region, *base* and *InBetween* location, this is done because these location object types can have both a strand position and an index position on the sequence. (*ii*) The *reference* property is not part of a Position, but of a Location, because a location that starts on one sequence and ends on another sequence is an undefined sequence. (*iii*) The *BaseLocation* and the *InBetweenLocation* classes have been added to the ontology. (*iv*) The *BaseLocation*, *InBetweenLocation*, *CollectionOfRegions* and *Region* are children of the *Location* class, such that the rest of the ontology can incorporate these classes. (*v*) The *before* and *after* positions have been explicitly defined to include their semantics. (*vi*) The classes sub-classed from *FuzzyPosition* have an integer to denote the position and do not point to another position object, which could allow for arbitrary complex location denotations. (*vii*) The N- and C-terminal positions have been removed and all indexes are counted from the N-terminal side. Counting from the C-terminal side can be calculated based on the sequence length. (*viii*) The reflective properties *beginOf* and *endOf* have been removed, because a position can also be referenced by the added base location. For consistency we have redefined all FALDO elements within our own namespace.

Over 350 cross-links to exact matching terms from other ontologies (such as FALDO, SO, SBOL or Wikidata) were added using *skos:exactMatch*. Additionally, several properties within the ontology point to existing ontologies, for instance: (*i*) The *signalTarget* property of SignalPeptide, the *modificationFunction* of *ModifiedResidue* and the *organelle* of *Sample* are interlinked with GO terms. (*ii*) The *experiment* property of ProvenanceAnnotation, which denotes upon which evidence the annotation is based on, should point, where possible, to a term within the Evidence Ontology. (*iii*) The *residue* property of *ModifiedResidue* must point to a term within the Protein Modification Ontology^47^. (*iv*) GBOL includes the GO terms for *tissueType* of the Sample class and points, when possible, to a term within the BRENDA Tissue and Enzyme Source Ontology^48,49^.

## Data Availability

Data sharing not applicable to this article as no datasets were generated or analysed during the current study.

## Code availability

Empusa is written in Java with Gradle as build system. Empusa code base is available at http://www.gitlab.com/Empusa under the MIT license. Documentation and tutorials can be found at associated website http://empusa.org.

Regarding GBOL, the source file of the ontology encoded in the Empusa and associated generated OWL definition (Passes OWL validation according to the HermiT Reasoner), ShEx schema and visualization for Cytoscape are available at http://www.gitlab.com/GBOL under the MIT license. The generated Java and R API are available at https://gitlab.com/gbol/GBOLapi and https://gitlab.com/gbol/RGBOLApi under the MIT license. Tutorials on using the Java and R API are available at http://gbol.life/APIUsage/. Instructions on how to perform a ShEx validation, with passing and failing examples are to be found at http://gbol.life/APIUsage/. All terms in GBOL are resolvable and can be browsed at the associated website http://gbol.life.
